# Trichostatin A treatment of cloned mouse embryos improves constitutive heterochromatin remodeling as well as developmental potential to term

**DOI:** 10.1186/1471-213X-9-11

**Published:** 2009-02-11

**Authors:** Walid E Maalouf, Zichuan Liu, Vincent Brochard, Jean-Paul Renard, Pascale Debey, Nathalie Beaujean, Daniele Zink

**Affiliations:** 1 INRA, UMR 1198 Biologie du Développement et Reproduction, F-78350 Jouy en Josas, France; 2 Department Biologie II, LMU München, Grosshaderner Str. 2, 82152 Planegg-Martinsried, Germany; 3 QMRI, 47 Little France Crescent, University of Edinburgh, Edinburgh, UK; 4 State Key Lab of Reproductive Biology, Institute of Zoology, Chinese Academy of Sciences, Beijing 100080, PR China; 5 UMR 5153 CNRS MNHN, Muséum national d'Histoire naturelle, CP26, 57 Rue Cuvier, 75231, Paris Cedex 05, France; 6 Institute of Bioengineering and Nanotechnology, 31 Biopolis Way, The Nanos, #04-01, Singapore 138669

## Abstract

**Background:**

Genome reprogramming in early mouse embryos is associated with nuclear reorganization and particular features such as the peculiar distribution of centromeric and pericentric heterochromatin during the first developmental stage. This zygote-specific heterochromatin organization could be observed both in maternal and paternal pronuclei after natural fertilization as well as in embryonic stem (ES) cell nuclei after nuclear transfer suggesting that this particular type of nuclear organization was essential for embryonic reprogramming and subsequent development.

**Results:**

Here, we show that remodeling into a zygotic-like organization also occurs after somatic cell nuclear transfer (SCNT), supporting the hypothesis that reorganization of constitutive heterochromatin occurs regardless of the source and differentiation state of the starting material. However, abnormal nuclear remodeling was frequently observed after SCNT, in association with low developmental efficiency. When transient treatment with the histone deacetylase inhibitor trichostatin A (TSA) was tested, we observed improved nuclear remodeling in 1-cell SCNT embryos that correlated with improved rates of embryonic development at subsequent stages.

**Conclusion:**

Together, the results suggest that proper organization of constitutive heterochromatin in early embryos is involved in the initial developmental steps and might have long term consequences, especially in cloning procedures.

## Background

The concept of cloning by nuclear transfer (NT) was introduced almost a century ago by Hans Spemann [1]. Central in NT experiments is developmental reprogramming of the donor nucleus after transfer into an enucleated recipient oocyte or zygote. A breakthrough in NT experiments was the birth of the first clone from a somatic cell in 1997 [2]. Today, a number of animal species have been cloned, but the success rate rarely exceeds 5% [3]. The success rate depends on technical skills and the biological material used. For example, embryonic stem (ES) cells are less differentiated and appear to be more efficiently reprogrammed when used as donor cells compared to somatic cells [4].

Many recent studies also suggested that nuclear remodeling after transfer might be involved in this reprogramming efficiency (for review see [5]). Indeed, in the early hours after natural fertilization, nuclear reorganization in embryos is associated with important modifications of paternal and maternal chromatin at the molecular and structural level. In most mammals, the protamines are rapidly replaced by histones in the paternal genome. Concurrently, the paternal pronucleus is demethylated to an extend that depend on the species [6], while the maternal pronucleus is passively demethylated upon several cell cycles [7,8]. Microscopically visible changes in the embryo include expansion of the pronuclei and the formation of the nucleolar precursor bodies (NPBs) [9]. Intriguingly, it has been shown in mouse embryos that centromeres and pericentric heterochromatin regions of chromosomes associate with the periphery of NPBs [10-12]. This characteristic zygotic organization of constitutive heterochromatin can be observed in 1-cell mouse embryos but not at later stages of development, nor in differentiated somatic cells [10,13]. In interphasic somatic cells these regions, essential for proper chromosome segregation [14], are usually forming chromocenters. Chromocenters indeed represent clusters of the pericentric regions of different chromosomes, surrounded by the centromeres of the corresponding chromosomes [15,16].

Interestingly, we and others previously showed that donor cell nuclei, with distinct chromocenters, can be remodeled into a zygotic-like heterochromatin organization after NT into enucleated oocytes [17,18]. However, this remodeling was often incomplete: a large proportion of the cloned embryos displayed, at the end of the first cell stage, an abnormally high number of centromeres not associated with NPBs, as compared to embryos obtained by natural fertilization at equivalent time-points. Interestingly, we showed that the percentage of embryos displaying this abnormal distribution of centromeres correlated with the percentage of ES cell-derived embryos that failed to develop until the blastocyst stage [17]. These findings supported the idea that proper genome remodeling in early embryos is essential for subsequent development.

On the other hand, it was recently shown that transient treatment of 1-cell stage mouse embryos with trichostatin A (TSA), a histone deacetylase inhibitor affecting chromatin structure, can significantly improve cloning efficiency after nuclear transfer from somatic cells [19,20]. In order to further address the hypothesis that proper nuclear remodeling in early embryos after nuclear transfer is essential, we investigated remodeling of centromeric and pericentric heterochromatin in embryos obtained by somatic cell nuclear transfer (SCNT) with or without TSA treatment. The results were further correlated with the developmental potential of the SCNT-derived embryos both *in vitro *(to the blastocyst stage) and *in vivo *(to term).

Here we confirm that in SCNT-derived embryos nuclear reorganization of centromeric/pericentromeric sequences also occurs but is often abnormal. Furthermore, the results show that nuclear remodeling is improved by transient TSA treatment, and that it was correlated with subsequent development of the embryos into blastocysts and healthy offspring. Together, the results suggest that genome remodeling into a zygotic-like organization is associated with the initial steps of embryonic development and that proper initial nuclear remodeling is essential for subsequent development.

## Results

### Nuclear organization is aberrant in 50% of SCNT embryos during the first cell cycle

**Figure 1:**
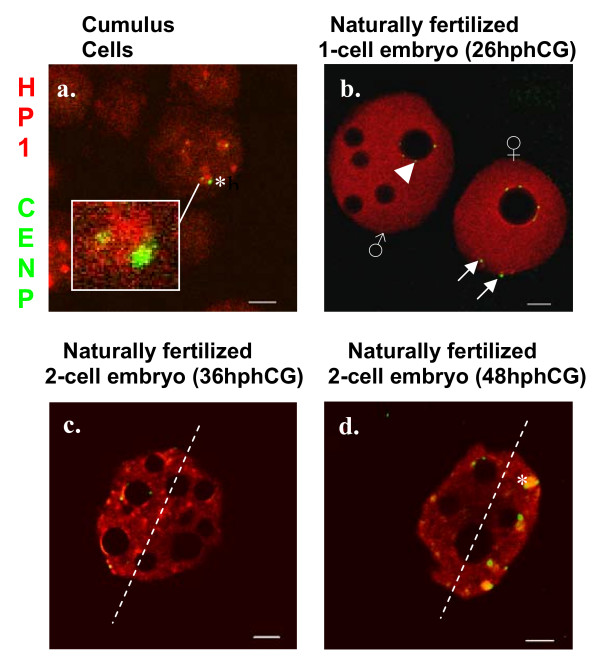
**Single light-optical sections showing the distribution of HP1β (red) and centromeres (green). **(a) mouse cumulus cell nuclei before transfer into an oocyte. (b-d) naturally fertilized embryos at 26 hphCG (b, late 1-cell), 36 (c, early 2-cell) and 48 hphCG (d, late 2-cell). Note the presence of chromocenters in panels a (* and insert) and d (*); whereas in 1-cell embryos (b), HP1β is essentially enriched at the NPB periphery of the female pronuclei. At that stage, most centromeres are localized at NPBs periphery (e.g. the centromeres underlined by the arrowhead), but some are excluded (arrows). In panels c and d, the dashed line delineates the nuclear halves either enriched in or devoided of chromocenters. Scale bars, 5 μm.

**Figure 2:**
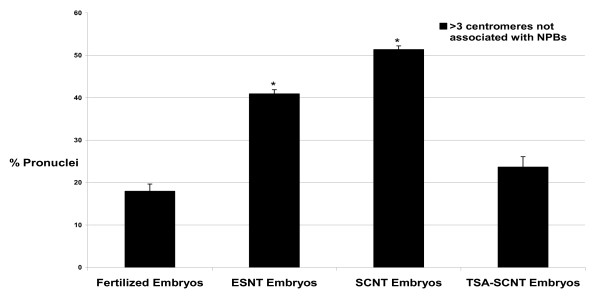
**Percentages of embryos displaying pronuclei with more than 3 centromeres not associated with NPBs**. Embryos were assessed at the late 1-cell stage: 26 hphCG for fertilized embryos, 10 hpa for ESNT and SCNT embryos (with or without transient TSA treatment). Groups labeled with an asterisk are statistically different from the "fertilized embryos" group (p < 0.05).

The zygote-specific nuclear organization observed during the first cell cycle of mouse embryos obtained by natural fertilization is characterized by the association of centromeres with the peripheries of nucleolar precursor bodies (NPBs, Figure 1b-arrowhead). We previously showed that about 80% of embryos obtained by natural fertilization display at most 3 centromeres not associated with NPB peripheries (Figure 2, [17]). Interestingly, this percentage is significantly decreased in 1-cell embryos produced by nuclear transfer of ES cells (ESNT) (~60%, p < 0.05) and correlates with the percentage of embryos that fail to develop into blastocysts [17]. In order to further address the relationship between nuclear remodeling and developmental potential, we investigated embryos produced by nuclear transfer of cumulus cells (SCNT), and analyzed the distribution of centromeric and pericentric heterochromatin during the first cell cycles. We therefore used immunofluorescent detection of heterochromatin protein 1β (HP1β), strongly enriched at pericentric heterochromatin, and of CENP proteins localized within the centromeres.

As expected for somatic mouse cell nuclei [15,16], constitutive heterochromatin in cumulus cells nuclei before SCNT is clustered into chromocenters (Figure 1a) that can easily be detected by the enrichments of HP1β into foci highlighted by the presence of associated centromeres (Figure 1a – insert).

**Figure 3:**
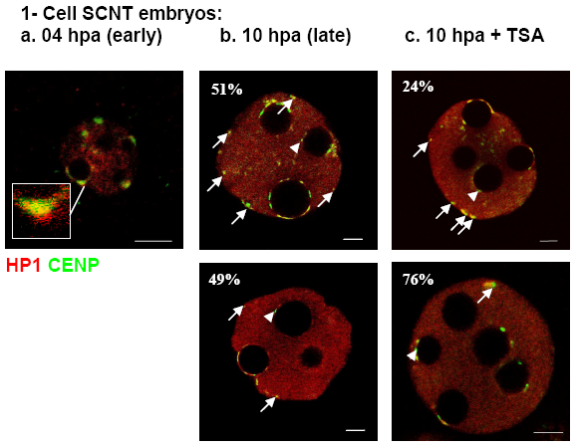
**Single light-optical sections showing the distribution of HP1β (red) and centromeres (green) in early (a, 4 hpa) and late (b-c, 10 hpa) 1-cell SCNT embryos, with or without TSA treatment**. At 4 hpa (a) centromeres are decondensed and mainly localized at NPB peripheries, occasionally associated with HP1β accumulations (insert). At 10 hpa (b-c) centromeres are more condensed and located at NPB (arrowheads) or nuclear peripheries (arrows). However, two types of embryos can be distinguished: the first one (b and c, top row) harboring more than 3 centromeres at the nuclear periphery, not associated to NPBs (as in 51% of the untreated embryos) whereas the second one (b and c, bottom row) exhibit 3 or less centromeres not associated with the NPBs (as in 76% of the embryos after transient treatment with TSA). Scale bars, 5 μm.

After SCNT, development of the embryos was initiated by artificial activation of the recipient oocytes in the presence of strontium. SCNT embryos were then analyzed during the 1-cell stage at 4 and 10 hours post activation (hpa). Already at 4 hpa donor nuclei derived from cumulus cells displayed substantial remodeling. NPBs, which are a characteristic component of early embryonic nuclei, but are not found in somatic nuclei, could already be observed (on average six per nucleus, n = 28, Figure 3a). Moreover, chromocenters were disrupted and the characteristic prominent foci of HP1β surrounded by defined dot-like centromeres could not be observed anymore. Instead, centromeres in such remodeled nuclei were decondensed as revealed by their diffuse appearance (Figure 3a), and mostly associated with NPB peripheries. HP1β displayed a relatively uniform nuclear distribution, although occasional accumulations of HP1β could be observed at NPB peripheries in close proximity to centromeres (Figure 3a – insert).

At 10 hpa, the pronuclei of SCNT embryos had increased in size and had a wider diameter than pronuclei at 4 hpa (Figure 3a/b–c, note scale bars differences). The numbers of NPBs did not change significantly compared to 4 hpa (n = 36, *p *= 0.85). At 10 hpa, the centromeres were not decondensed anymore and appeared again as small well defined dots, mostly located at NPBs peripheries. HP1β was also enriched at NPBs peripheries and confined to a thin rim encircling NPBs (Figure 3b). As outlined above, such organization of centromeres is a key feature of 1-cell stage embryos. Moreover, the rim distribution of HP1β is similar to the one typically observed during the 1-cell stage in female pronuclei of fertilized embryos (at this stage the male pronucleus essentially displays a diffuse HP1β labeling without visible accumulations, Figure 1b). These results suggest a strong remodeling of cumulus cell nuclei after NT. However, half of the SCNT embryos we investigated harbored pronuclei where more than 3 centromeres were not associated with NPBs (51%, Figures 2 and 3b), a proportion slightly but not significantly higher than in ES-cell derived clones (41%, n = 22, *p *= 0.54).

**Figure 4:**
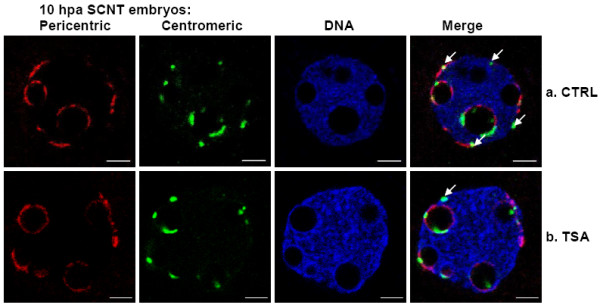
**Three-dimensional fluorescent in situ hybridization (3D FISH) performed on 1-cell SCNT embryos that developed until 10 hpa in the absence (a. CTRL) or presence (b. TSA) of TSA**. FISH signals of pericentric and centromeric repeats are displayed in red and green, respectively, with DNA counterstaining in blue. Note the close but distinct apposition of the centromeric and pericentric signals. Arrows point to centromeres not associated with NPB peripheries. Scale bars, 5 μm.

As immunodetection might be affected by the presence/absence of the antigens we then confirmed our observations with three-dimensional fluorescence *in situ *hybridization (3D FISH) using probes specific for centromeric and pericentric sequences. Results showed that, at 10 hpa, centromeres and pericentric sequences are indeed not clustered in nucleoplasmic foci. Instead, these sequences are associated with the nuclear and NPB peripheries (Figure 4a). Remarkably, in 46% of the clones (n = 13) a high proportion of these sequences were clearly observed at the nuclear periphery. These results are in accordance with our data obtained by immunofluorescent detection.

Altogether, our data show that constitutive heterochromatin, especially the prominent chromocenters present in somatic cell nuclei, is rapidly remodeled into a zygotic-like organization during the 1-cell stage of embryonic development, regardless of the starting material (ES or somatic cell). However, we also often observed aberrant reorganization of the centromeres that frequently did not associate with NPB peripheries but with the nuclear periphery.

### Nuclear rearrangements are similar in 2-cell SCNT and fertilized embryos but quantitative differences are observed

**Figure 5:**
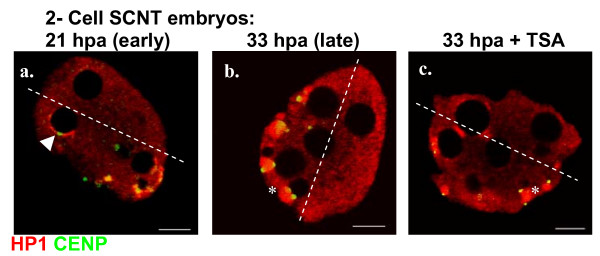
**Single light-optical sections showing the distribution of HP1β (red) and centromeres (green) in 2-cell SCNT embryos at 21 hpa (a, early) and 33 hpa (b-c, late), with or without TSA treatment**. The arrowhead in panel a points to a centromere associated with a NPB. Asterisks in panels b and c label chromocenters. Dashed lines delineate nuclear halves either enriched in or devoided of centromeres and chromocenters. Scale bars, 5 μm.

During the second cell cycle, embryos obtained by natural fertilization display dramatic nuclear rearrangements and relocalization of centromeric and pericentric heterochromatin. These dynamic events are associated with the rapid formation of chromocenters [10]. Here, we observed similar nuclear rearrangements and rapid formation of chromocenters in SCNT embryos during the second cell cycle. In early 2-cell embryos (21 hpa) most centromeres were still associated with NPB peripheries (n = 20, Figure 5a – arrowhead). In contrast, at the end of the 2-cell stage (33 hpa), centromeres were associated to chromocenters displaying the characteristic enrichment in HP1β (n = 48, Figure 5b – asterisk). In addition to chromocenter formation, we observed in SCNT 2-cell stage embryos a preferential localization of centromeres and chromocenters in one half of the nuclei (Figures 5a–c). A similar reorganization between the beginning and the end of the 2-cell stage, with a Rabl-like polarization was previously observed in fertilized embryos (Figure 1b–c, [12]), ES-cells derived embryos [17], and SCNT embryos [18]. However, at the end of the 2-cell stage, when comparing SCNT (33 hpa, n = 48) and fertilized embryos (48 hphCG, n = 20), we noticed that the number of chromocenters as well as the number of NPBs was lower in SCNT embryos (7.3 ± 2.6 and 6. 5 ± 2.7 respectively) than in fertilized ones (8.7 ± 2.3, *p *= 0.044 and 8.9 ± 2.2, *p *= 0.001 respectively).

In conclusion, although similar nuclear rearrangements occur during the 2-cell stage in SCNT and fertilized embryos, significant quantitative differences are observed that might affect the onset of genome expression taking place at that time.

### TSA improves nuclear reorganization of SCNT embryos during the first and second cell cycle

It was recently shown that transient treatment of mouse 1-cell stage embryos with TSA significantly improves the success rate of cloning with cumulus cells [19,20]. TSA is known to increase histone acetylation in both somatic cells and embryos [20-22]. In addition, TSA has also been reported to induce extensive reorganization of pericentric heterochromatin and centromeres in somatic mammalian cell nuclei [23-25]. We therefore questioned whether TSA treatment on early SCNT embryos might improve constitutive heterochromatin reorganization and how this could be correlated to the developmental rate. We chose to treat the cumulus derived SCNT embryos with the optimal conditions reported by the aforementioned authors, i.e. 5 nM for 10 h from the time of activation but in a different standard culture media, frequently used in our laboratory (M16 instead of KSOM). On these embryos fixed at various time points during the 1- and 2-cell stages, we first checked that the TSA treatment enhanced the histone acetylation level. Indeed, acetylated histone H4 K5 is known to increase significantly at the perinuclear region after TSA treatment [23-25]. We then performed immunofluorescent analysis with CENP/HP1β antibodies at the same time points as above.

When SCNT embryos developed in the presence of TSA the distribution of centromeres and pericentric heterochromatin at 4 hpa were similar to untreated SCNT embryos (n = 22, data not shown). At 10 hpa, however, a significantly lower number of embryos displayed aberrant nuclear remodeling: the frequency of embryos harboring pronuclei with 3 or more centromeres not associated with NPBs decreased from 51% (n = 36) in untreated to 24% (n = 46) in TSA-treated SCNT embryos (Figures 2, 3c, 4b). This percentage was not significantly different from the one observed in fertilized embryos (18%, n = 39, *p *= 0.196) at an equivalent time-point (25 hphCG).

We next investigated the effects of TSA treatment on nuclear rearrangement events taking place during the 2-cell stage. At 33 hpa, we found that TSA had no effect on chromocenter formation and the number of chromocenters formed in TSA-treated SCNT embryos remained significantly lower (6.7 ± 3.3, n = 56) than in fertilized embryos (8.7 ± 2.3, n = 20, *p *= 0.04). However, there was an increased number of NPBs in TSA-treated SCNT embryos (8.6 ± 2.9, n = 56) in comparison to untreated ones (6.5 ± 2.7, n = 48, *p *= 0.002). Remarkably, after TSA treatment the number of NPBs observed in SCNT embryos was similar to the one observed in fertilized embryos (8.9 ± 2.2, n = 20).

Together, these findings show that TSA treatment improves the remodeling of transferred nuclei both at the 1-cell stage with the acquisition of a zygotic-like heterochromatin organization and at the 2-cell stage with the rearrangements of NPBs.

### TSA improves further development of SCNT embryos

**Figure 6:**
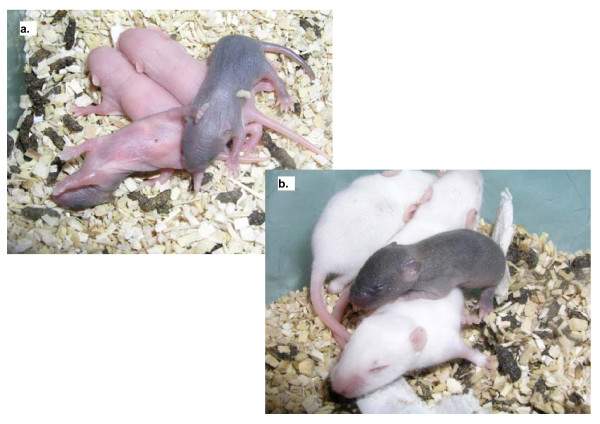
**Live pup obtained after somatic cell nuclear transfer and transient treatment with 5 nM TSA (brown fur), with control foster brothers (white fur), 2 (a) and 6 (b) days after caesarian section**.

Next, we examined the effects of TSA treatment on further embryonic development. The rates of activation and cleavage to the 2-cell stage were similar in TSA-treated and untreated SCNT embryos (Table 1). The rate of development to blastocysts was not statistically different after TSA treatment (Table 1, 35% with vs 31% without TSA, p = 0.5). However, blastocysts from the TSA-treated group had a greater number of inner cell mass (ICM) cells than those from the untreated controls (10.4 ± 2.9 vs 13.9 ± 4.2, *p *= 0.03), albeit the total cell numbers were not different (data not shown). More interestingly, the number of live pups obtained with TSA-treated SCNT embryos (Figure 6) was significantly higher than with non-treated ones (3.1 vs 0.3%, *p *= 0.007, Table 1). Thus improved foetal development and increased numbers of live offspring can be correlated with transient TSA treatment during early development of SCNT embryos and improved nuclear remodeling at the initial stages of development.

**Table 1:** The developmental rates of somatic cell nuclear transfer (SCNT) embryos in the absence or presence of TSA.

	**N° activated**	**N° at 2c (%)**	**N° transferred**	**N° live offspring (%)**	**N° 2c remaining in culture**	**N° at blastocyst (%)**
**SCNT Embryos**	669	568 (85)	342	1 (0.3)	226	71 (31.4)

**TSA-SCNT Embryos**	387	319 (82.5)	193	6 (3.1)	126	44 (34.9)

Statistical difference was calculated using Chi-square and was found to be significant for live offspring only (*p*-value < 0.01).

## Discussion

Kishigami and colleagues [19] were the first to report that TSA treatment improves full term development of mouse embryos obtained by transfer of cumulus cell nuclei. This was confirmed the same year by Rybouchkin and colleagues who reported a remarkable and significant 5-fold increase in the efficiency of cloning from cumulus cells with a transient TSA treatment for 10 hours post activation [20]. In their initial work, Rybouchkin and colleagues suggested that increased acetylation of histones after TSA treatment was linked to the improved developmental rates [20]. In the present work, we confirm the reproducibility of the beneficial effects of TSA treatment on long term developmental potential using another mouse strain and different culture conditions, as reflected by a significantly higher birth rate of live pups. In our laboratory, 3.1% of the TSA-treated SCNT embryos developed to term, which is identical to the 3.1% of clones obtained from ES cells [26] and ten times higher than the developmental rate of control SCNT embryos (0.3%).

However, TSA was also reported to affect chromatin condensation and relocation thereby affecting higher order nuclear organization during interphase [23-25]. We therefore focused the present work on the effects of transient TSA treatment upon heterochromatin remodeling events during the first two cell cycles after nuclear transfer of cumulus cell nuclei, based on previous reports of constitutive heterochromatin distribution in early mouse embryos after fertilization and/or nuclear transfer [10,11,17,18].

So far, it has been shown that constitutive heterochromatin becomes remodeled into a zygote-specific organization during the 1-cell stage of embryonic development after fertilization as well as after ESNT and cumulus SCNT [10,11,17,18]. As sperm, oocytes, ES and cumulus cells display very different forms of chromatin organization prior to their introduction into an oocyte, these findings suggested that remodeling of chromatin into this particular form of organization might be fundamentally associated with the initial stages of development and might always occur, regardless of the starting material. Here, we confirm that nuclear reorganization occurs after transfer of differentiated cumulus nuclei into an oocyte. We also show that this remodeling process was very rapid (at 4 hpa chromocenters disruption already occurred as well as NPBs formation). Remodeling was, however, not efficient in all nuclei, since about half of them showed a higher number of centromeres not associated with NPBs.

The main finding is that TSA treatment largely improves the efficiency of this initial remodeling, since the percentage of transferred nuclei displaying a configuration with few mis-located centromeres is the same in TSA treated clones and in embryos from natural fertilization. The supplementation of TSA during the first cell cycle also affects the nuclear organization of embryos during the second cell cycle and increases the average number of NPBs in SCNT treated embryos (vs. non-treated ones), to the same level as in fertilized ones. This differs from the results of Merico and colleagues [18], where similar numbers of NPBs in late 2-cell SCNT and control embryos were reported. However, in that case in vitro fertilized embryos were used as controls, as well as a different strain of mice (C57/CBAF1 vs C57/C3HF1). What should be noted is that defects in the number and distribution of NPBs may be correlated with delayed acquisition of functional nucleoli in cloned embryos [27] and that TSA treatment might improve it. However, the exact mechanism through which TSA improves nuclear remodeling is still unclear. One could hypothesize that the increase in histone acetylation induced by TSA improves "opening" of the chromatin thereby sustaining mobility and relocalization of constitutive heterochromatin (normally characterized by hypoacetylation and HP1β binding) as well as other genomic sequences (such as nucleolar organizing regions maybe). Indeed, 3D-FISH measurements of the volume occupied by pericentric sequences in TSA-treated and untreated SCNT embryos suggest a slight increase upon TSA treatment: in untreated embryos pericentric chromatin occupies 2.05% of the nucleus versus 2.18% in TSA-treated embryos (p < 0.5, Man and Whitney test, n = 18 & 21 respectively). One could also hypothetize that chromatin decondensation after TSA treatment and histone acetylation will allow access of different remodeling factors from the ooplasm. Further work investigating the functional relationship between genome reprogramming and nuclear reorganization is currently under way in our group.

On the other hand, it is worth mentioning that the effects of TSA are not universal and are highly dependent on the level of differentiation of the genome treated. Somatic and ES cells, for example, have different gene expression and epigenomic profiles, including the CpG methylation levels at the centromeric and pericentric regions [28]. In a supplementary experiment, we tested transient TSA treatment on ES-cells derived NT and observed that TSA actually raises from 40% up to 62% the fraction of pronuclei presenting more than 3 centromeres not associated with NPBs by 10 hpa. Moreover, no live pup was reported after such treatment ([19] and our data not shown). This suggests that TSA is not always beneficial and that the balance of acetylation and deacetylation activities should be maintained. Trichostatin A probably exacerbates the profile of some key genes in early ESNT embryos while improving their profile of gene expression in SCNT embryos. Whether gene expression is a cause or consequence of genome reorganization remains to be determined.

## Conclusion

The findings of the present study support the idea that initial nuclear reorganization plays an important role in genome reprogramming and is thus also important for further development. Current models of nuclear architecture acknowledge that the positioning of gene loci at specific regions of the nucleus affects their expression and contributes to gene regulation [29-35]. We therefore believe that TSA-induced improvement of nuclear remodeling might support a more accurate regulation of developmental genes, crucial at the beginning of development. However, the very low birth rate, even after TSA treatment, implies that the nuclear reorganizations observed are important but often not sufficient for later development. To address this problem, further investigations of more subtle modifications in the nuclear remodeling processes and in chromatin structure after nuclear transfer are necessary.

## Methods

All experiments involving animals were carried out according to European regulations on animal welfare.

### Mouse Embryo Collection and Culture

Oocytes were prepared by superovulating C57/CBA mice. Superovulation was induced by injecting pregnant mare serum gonadotropin (PMSG, Intervet, 5 UI) and human chorionic gonadotropin (hCG, Intervet, 5 UI) at intervals of 48 hours. Oocytes were collected from oviducts 14 hphCG (hours after injection of hCG) and washed in M2 (Sigma) medium containing 1 mg/ml hyaluronidase. Subsequently, they were incubated in M2 containing 5 μg/ml cytochalasin B and placed in a chamber on the stage of an inverted microscope (Nikon) equipped with micromanipulators (Nikon-Narishige MO-188). The chromatin spindle (visualized under differential interference contrast) was aspirated into the pipette as described by [26]. For SCNT, donor chromosomes were derived from cumulus cells that previously surrounded the oocytes, gently aspirating them in and out of the injection pipette (inner diameter 7–8 μm) followed by microinjection into the cytoplasm of the enucleated oocytes. The nuclear transfer embryos were activated by incubation for 6 h in Ca^2+^-free medium containing 10 mM Sr^2+^, 5 μg/ml cytochalasin B (CB), and in the presence or absence of 5 nM trichostatin A (TSA).

Embryos with visible nuclei were considered as activated, were transferred into fresh M16 medium and cultured at 37°C in a humidified atmosphere containing 5% CO_2_. For TSA treatment embryos were cultivated for another 4 hours in M16 supplemented with 5 nM TSA before in vitro culture in M16 medium without supplements. Embryos were fixed during the first cell cycle at 4 hours post-activation (4 hpa), 10 hpa, and early and late 2-cell stages (21 hpa and 33 hpa respectively). In vitro culture was carried out in M16 medium (Sigma) at 37°C in a humidified atmosphere containing 5% CO_2_. For naturally fertilized embryos, superovulated females were mated with male mice at the time of hCG injection. Collection and culture of those embryos were carried out similarly to that of SCNT embryos. All experimental sets contained embryos from different mice taking the relative asynchrony of fertilization into consideration.

### Immunofluorescent detection and mounting

Embryos were fixed with 2% paraformaldehyde (PFA) in PBS for 30 min at room temperature (RT) and permeabilized with 0.5% Triton X-100 (30 min, RT). For immunostaining, embryos were blocked with 2% bovine serum albumin (BSA) in PBS for 1 hour. Incubation with the primary antibodies was performed overnight at 4°C. After two washes with PBS, embryos were incubated with the secondary antibodies (1 h at RT) and rinsed again in PBS to remove excess of antibodies. All antibodies were diluted in PBS-BSA (2%). The mouse monoclonal anti-HP1β antibody was obtained from Euromedex (clone 1MOD1A9, 1:400). The centromeres were labeled with a human CREST antibody which recognizes both CENP-A and B (HCT-0100, Immunovision, Springdale, AR, 1:400). The fluorescently labelled secondary antibodies were purchased from Jackson Immunoresearch in West Grove, PA, and used at a dilution of 1:400. Embryos were then briefly post-fixed (2% PFA, 10 min, RT), and embryos were deposited on depressed slides (for 3D preservation) and mounted under a coverslip with Citifluor (Citifluor Products).

### Three dimensional-fluorescent in-situ hybridization

Embryos had their zona pellucida removed in Tyrode solution, fixed in 4% PFA for 30 min at RT, and then deposited on a slide. Subsequently, the embryos were permeabilised in 0.5% Triton X-100 for 30 min at RT, and treated with RNAse for another 30 min at 37°C. The oligonucleotides for hybridization were prepared by amplifying the regions that correspond to the centromeric using 5'-ACTCATCTAATATGTTCTACAGRG-3' and 5'-AAAACACATTCGTTGGAAAC GGG-3' primers, and the pan-centromeric region using the 5'-CATATTCCAGGTCCTTCAGTGTGC-3' and 5'-CACTTTAGGACGTGAAATAT GGCG-3' primers (provided by H. Masumoto, Nagoya University, Japan) from mouse genomic DNA. The corresponding probes were then labeled with the Bioprime Array CGH genomic labeling system (Invitrogen, USA). After a number of washing steps in hybridization buffer, embryos and pan-centromeric and centromeric probes were denatured at 85°C, and then they were hybridized together at 37°C. After 24 hours of hybridization, the DNA was counterstained with YoPro I for 15 min at RT, before adding Citifluor (Citifluor Products) and the coverslip.

### Microscopy and image analysis

Confocal microscopy was performed with a Zeiss LSM 510 confocal laser scanning microscope equipped with an oil immersion objective (Plan Apochromatic 63× n.a.1.4), with the 488-, 535-, and 633-nm wavelengths lasers. Entire embryos were scanned with a distance between light optical sections ranging from 0.3 and 0.4 μm. 3D recontructions of image stacks and image analysis were performed using LSM 5 browser and ImageJ software.

### Statistical Analysis

More than 20 embryos were examined in every group, and each experiment was repeated at least 3 times. For testing the significance of differences the Chi-square test and the Student T-test were used applying corresponding functions of MS-Excel (2002) software. Differences were assumed to be significant at *p*-values < 0.05.

## Authors' contributions

WM has planned the experiments, analysed the data and also drafted the manuscript. WM, ZL and VB have contributed to the execution of the experiments. NB, PD, JPR and DZ conceived the overall project, designed and coordinated the work, and also participated in the drafting of the manuscript. All Authors have read and approved the manuscript.
